# Does the short-term learning effect impact vertical jump performance assessment on a portable force plate system?

**DOI:** 10.3389/fspor.2024.1441022

**Published:** 2024-08-12

**Authors:** Damjana V. Cabarkapa, Dimitrije Cabarkapa, Jelena Aleksic, Igor Ranisavljev, Andrew C. Fry

**Affiliations:** ^1^ Jayhawk Athletic Performance Laboratory—Wu Tsai Human Performance Alliance, Department of Health, Sport and Exercise Sciences, University of Kansas, Lawrence, KS, United States; ^2^ Faculty of Sport and Physical Education, University of Belgrade, Belgrade, Serbia

**Keywords:** force, power, testing, monitoring, athlete, eccentric, concentric, neuromuscular

## Abstract

One of the reoccurring questions that arises during the countermovement vertical jump (CVJ) assessment is whether the learning effect has an impact on the accuracy of the results obtained. Thus, the purpose of the present investigation was to examine the impact of the short-term learning effect on the assessment of lower-body neuromuscular performance characteristics when performed on a portable one-dimensional force plate system. Sixteen recreationally active college-age males volunteered to participate in the present study. Each participant completed four sets of three non-consecutive CVJs with no arm swing throughout a single day. Besides strong verbal encouragement, participants were constantly instructed to focus on pushing the ground as explosively as possible. Fourteen force-time metrics were selected for CVJ performance analysis purposes: eccentric and concentric peak and mean force and power, eccentric and concentric duration, contraction time, jump height, reactive strength index-modified, and countermovement depth. Repeated measures multivariate analysis of variance was used to examine statistically significant differences across four testing time points (*p *< 0.05). The results indicate an absence of any meaningful differences across four testing time points in force-time metrics of interest during both eccentric and concentric phases of the CVJ. Moreover, no differences were observed in CVJ outcome metrics such as countermovement depth, suggesting that the movement strategy tends to remain consistent. Overall, these findings reveal that CVJ test repeatability is not affected by the short-term learning effect and that data are stable at least within the scope of this study and within this population.

## 1 Introduction

Over recent years, a substantial rise in professionalism across sports, such as advancements in sports science, technology, and education, has resulted in athletes being subjected to greater training volumes and frequencies throughout the competitive season (1). The aforementioned increase in the training load further supports the need for frequent athlete monitoring in order to help athletes optimize their performance and mitigate the adverse effects of neuromuscular fatigue (1).

Neuromuscular fatigue is a complex phenomenon often influenced by various factors such as the type and duration of exercise, as well as the speed and duration of muscle contraction (2, 3). It has been previously defined as “any exercise-induced reduction in force or power regardless of whether the task can be sustained or not (2, 4, 5)”. Regarding whether the reductions in muscle force and power originate from processes distal to the neuromuscular junction or within the motoneurons and central nervous system, neuromuscular fatigue can be categorized as peripheral or central, respectively (2, 4, 5).

One of the commonly used testing modalities for the assessment of neuromuscular fatigue is the countermovement vertical jump (CVJ) (6–8). While various technologies have been used for monitoring CVJ performance, force plate systems are still considered a gold standard testing modality (i.e., criterion measure) due to their accuracy and capacity to provide practitioners with numerous force-time metrics (6–8). However, one of the reoccurring questions that arises during the CVJ assessment performed on force plate systems is whether the learning effect has an impact on the accuracy of the results obtained during testing, especially when first introducing this test. Specifically, as athletes become more familiar with the CVJ testing protocol, would their performance considerably improve?

To the best of our knowledge, only a few research reports have focused on addressing the previously mentioned question and investigating the impact of familiarization and/or learning effects on athletes' CVJ performance (9–13). For example, Moir et al. (11) examined the number of familiarization sessions required before an accurate measure of reliability associated with unloaded and loaded CVJ performance and 10 and 20-meter sprint running times in physically active males. The authors showed that high reliability can be achieved in the CVJ and 10 and 20-meter sprint tests without the implementation of the familiarization sessions (11). Similar findings were observed by Nibali et al. (12) indicating that both male and female athletes do not require familiarization trials before CVJ assessment, regardless of the level of play (i.e., high school, college, and professional). However, Vrbnik et al. (13) revealed contradictory results, inferring that elementary school children significantly improved their CVJ and standing long jump performance from the first to penultimate and ultimate testing session, respectively (i.e., five total testing sessions). Lastly, when examining the presence of a learning effect on the Wingate anaerobic test, Barfield et al. (9) found that both peak and mean power significantly increased from the first (764.5 and 604.9 W, respectively) to the second testing session (867.6 and 634.7 W, respectively) in young adult males.

While the aforementioned findings provide insight into the impact of the learning effect and familiarization with testing processes on performance, they still remain inconclusive. Therefore, in order to bridge a gap in the scientific literature, the purpose of the present investigation was to examine the impact of the short-term learning effect on the assessment of lower-body neuromuscular performance characteristics when performed on a portable force plate system.

## 2 Materials and methods

### 2.1 Participants

Sixteen college-age males (age = 20.7 ± 1.8 years; height = 184.1 ± 6.7 cm; body mass = 83.1 ± 8.3 kg) volunteered to participate in the present study. All participants were free of any kind of musculoskeletal injuries that could impact CVJ performance, and they all actively participated in individual or team recreational activities 3–4 times per week >60 min (e.g., resistance exercise, pick-up basketball, tennis, boxing). The testing procedures performed were approved by the University's Institutional Review Board and all participants signed an informed consent document.

### 2.2 Procedures

Following completion of a standardized warm-up protocol consisting of a 5-min moderate-intensity jog and dynamic stretching exercises (e.g., A-skips, high-knee-pulls, karaoke, lunges, butt-kicks), all participants completed four sets of three non-consecutive CVJs. The participants were instructed to step on a portable uni-axial force plate system sampling at 1,000 Hz (ForceDecks Max, VALD Performance, Brisbane, Australia) and perform three maximum-effort CVJs while keeping their hands on the hips during the entire movement (i.e., CVJ with no arm swing). Specifically, participants were asked to get in a “ready position” (i.e., standing upright with feet shoulder width apart and looking straight ahead), and from this position, when the research assistant says “start”, begin a downward movement to a self-selected height and perform a maximal vertical jump by pushing the ground as explosively as possible (14). Each CVJ within a single testing time point was separated by a 15–20 s rest interval to minimize a possible impact of fatigue. The mean value across three jump trials within each testing time point was used for performance analysis procedures. All participants completed an additional three sets of three CVJs following the identical instructions and testing procedures. Each set of three CVJs was separated by a 15-minute rest interval. In addition, the force plate system was zeroed between each participant, and it was positioned on a solid surface (e.g., weightlifting room floor).

### 2.3 Variables

Fourteen force-time metrics were selected for CVJ performance analysis purposes based on previously published research reports, as variables that demonstrated considerable levels of validity and reliability when used in an applied sport setting (6, 7, 15–17). These variables of interest examined in the present study were eccentric and concentric peak and mean force and power, eccentric duration, contraction duration, jump height (i.e., impulse-momentum calculation), reactive strength index (RSI)-modified (i.e., jump height divided by contraction time), and countermovement depth. The start of the contraction time was determined when the system mass was reduced by 20 N and it ended when the vertical ground reaction force fell below the 20 N threshold. The eccentric phase was defined as the phase with a negative center of mass velocity (17–19).

### 2.4 Statistical analysis

Descriptive statistics, means and standard deviations $\left(\bar{x}\pm \mathrm{SD}\right)$, were calculated for each dependent variable. Shapiro-Wilk test and Q-Q plots were used to check that the assumption of normality was not violated. One-way repeated measures multivariate analysis of variance (MANOVA) was used to examine the statistically significant differences in the dependent variables across four testing time points. If needed, follow-up ANOVAs with Tukey post-hoc comparisons were used to examine the statistically significant main effects. Statistical significance was set *a priori* to *p *< 0.05. All statistical analysis procedures were completed in R software (Version 4.2.1; R Foundation for Statistical Computing, Vienna, Austria).

## 3 Results

**Figure 1:**
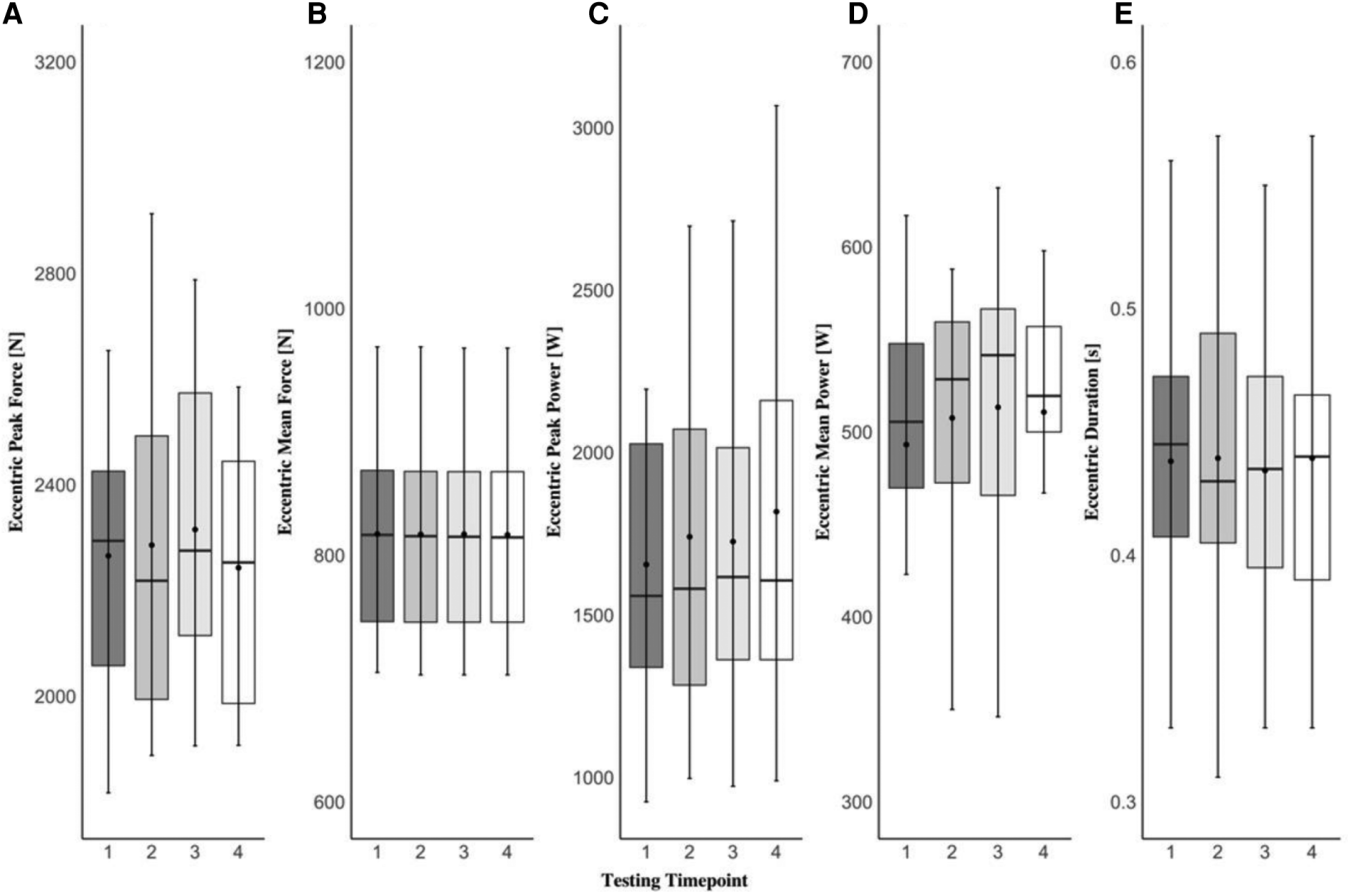
Countermovement vertical jump eccentric peak force (**A**), eccentric mean force (**B**), eccentric peak power (**C**), eccentric mean power (**D**), and eccentric duration (**E**) across four testing time points. The black dot resembles the mean value, the black line within the box plots represents the median value, the black dashes (whiskers) represent the minimum and maximum values, and the shaded areas represent interquartile ranges for each testing time point.

**Figure 2:**
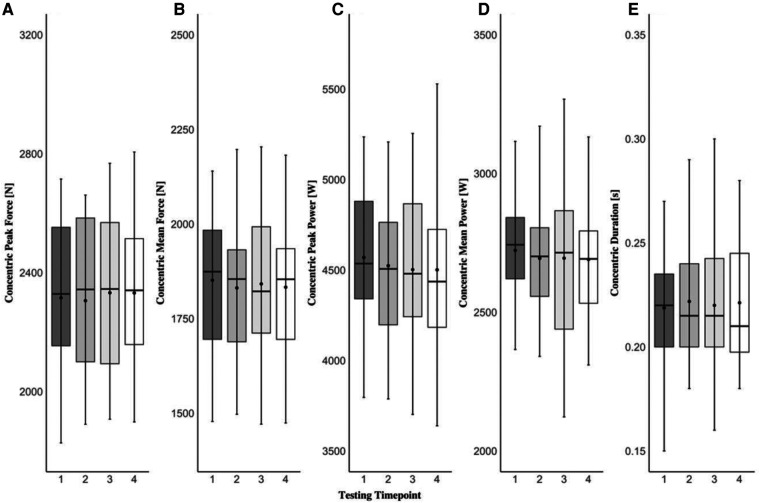
Countermovement vertical jump concentric peak force (**A**), concentric mean force (**B**), concentric peak power (**C**), concentric mean power (**D**), and concentric duration (**E**) across four testing time points. The black dot resembles the mean value, the black line within the box plots represents the median value, the black dashes (whiskers) represent the minimum and maximum values, and the shaded areas represent interquartile ranges for each testing time point.

**Figure 3:**
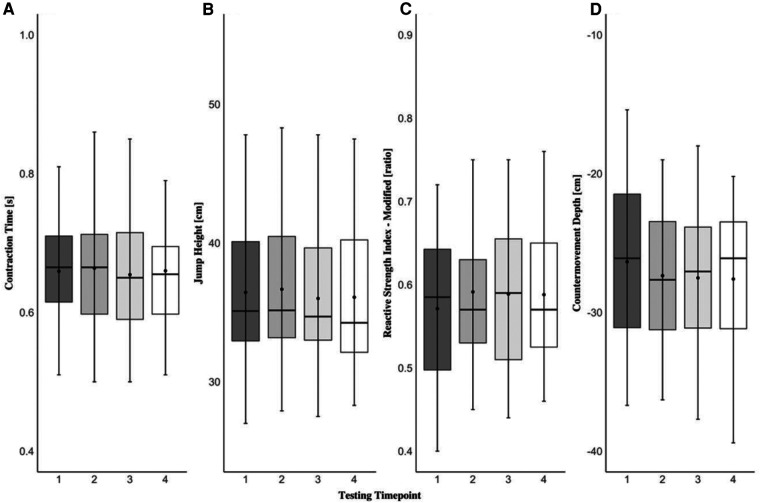
Countermovement vertical jump contraction time (**A**), jump height (**B**), reactive strength index-modified (**C**), and countermovement depth (**D**). The black dot resembles the mean value, the black line within the box plots represents the median value, the black dashes (whiskers) represent the minimum and maximum values, and the shaded areas represent interquartile ranges for each testing time point.

The one-way repeated measures MANOVA revealed that there were no statistically significant differences (F[3,45]_ _= 1.507; *p *= 0.061, Λ* *= 0.222, $\eta_{p}^{2}$^ ^= 0.395) across four testing time points in any force-time metrics examined in the present study during both eccentric and concentric phases of the CVJ movement. Specifically, none of the variables were affected by the short-term learning effect and the data remained stable within the scope of this study and this population. A detailed graphical representation of each dependent variable examined in the present study can be found in Figures 1–3.

## 4 Discussion

To the best of our knowledge, this is the first study focused on examining the impact of the short-term learning effect on the assessment of lower-body neuromuscular performance characteristics when performed on a portable force plate system. The results indicate an absence of any statistically significant differences across four testing time points in force-time metrics of interest during both eccentric and concentric phases of the CVJ (e.g., mean and peak force and power, contraction time, RSI-modified). Moreover, no significant differences were observed in CVJ outcome metrics such as countermovement depth and vertical jump height, suggesting that the movement strategy tends to remain consistent.

When examining a similar topic, previous research has found high reliability (ICC = 0.89–0.95 and CV = 1.9%–2.6%) in the CVJ and acceleration sprinting performance within a cohort of physically active males, implying that the aforementioned assessments can be performed without the familiarization sessions (11). These results are in line with our findings indicating that the learning effect does not significantly alter the CVJ performance of recreationally active males across four different testing time points. However, familiarization trials seemed to be necessary when examining maximum knee extension strength, peak and mean power during the Wingate anaerobic test, as well as bench press, squat, and arm curl one repetition maximum (i.e., 1RM) testing (9, 20, 21). For example, Ploutz-Snyder & Giamis (21) examined the number of familiarization sessions required for untrained older (66.6 ± 5.0 years) and younger (23.0 ± 4.0 years) female participants in order to achieve consistent 1RM. While both groups needed familiarization sessions, possibly due to the lack of previous resistance training experience, the older females required more practice trials when compared to the younger subjects with the same training competence to achieve constant 1RM for knee extension exercise (21). In addition, similar observations were made by Dias et al. (20) showing that 1RM bench press, squat, and arm curl significantly increased from the first to last testing time point (2.4%, 3.4%, and 5.4%, respectively) in male subjects with previous lifting experience, further implying the need for familiarization in order to obtain accurate 1RM estimation. Lastly, Barfield et al. (9) observed that the learning effect occurs during the Wingate anaerobic test in young adult males, therefore resulting in higher peak and mean power from the first (604.9 and 764.5 W, respectively) to last testing session (634.7 and 867.6 W, respectively). Thus, based on the aforementioned research reports, it can be implied that the necessity for familiarization in strength and power tests mainly depends on the essential motor skills required for the testing modality being performed (CVJ test vs. 1RM bench press) (11). The CVJ is commonly integrated into various physical activities throughout life (e.g., jumping over obstacles, jumping rope), compared to exercises such as bench press or knee extension. Hence, it is reasonable to observe significant learning effects occurring in assessments that require higher levels of technical proficiency (e.g., bench press) or familiarization compared to ones such as CVJ.

Furthermore, the previously mentioned absence of learning effect in the present investigation during the CVJ performance can be explained by the implementation of standardized and consistent cues provided by the researchers administering the study. Specifically, participants were asked to place their hands on the hips, assume a “ready position” (i.e., standing upright with feet shoulder width apart and looking straight ahead), and from this position, when the research assistant says “start”, begin a downward movement to a self-selected height and perform a maximal vertical jump by pushing the ground as explosively as possible (14, 22). Previous studies have highlighted the importance of consistent verbal cues during jumping movements (e.g., CVJ, drop jump) (14, 23, 24). For example, Barillas et al. (23) revealed that instructing participants to “try and touch the ceiling with their head” led to increased vertical jump displacement in both the CVJ and drop jump, while providing an external velocity cue such as “get off the ground as fast as you can” resulted in reduced ground contact time during both assessments. Similar findings were observed by Barker et al. (24) showing that cueing NCAA Division-I soccer athletes to “get off the ground as quickly as possible” significantly increased their RSI during the drop jump. In addition, Kersher et al. (14) showed that external focus instructions such as “concentrate on pushing away from the ground as explosively as possible” considerably increased vertical jump height, peak velocity, mean concentric power and velocity, mean eccentric rate of force development and relative concentric impulse compared to the condition when subjects were instructed to use internal focus cues such as “concentrate on extending your knees and hips as explosively as possible”. Therefore, these findings support that externally focused and consistent cues during CVJ assessments may help athletes optimize their jumping technique and maintain focus throughout multiple testing sessions, as well as help practitioners avoid instruction-induced variability in jumping performance.

While the findings of the present investigation provide valuable information regarding the impact of the short-term learning effect on CVJ performance when performed on a force plate system, this study is not without limitations. The group of participants involved in this investigation was homogeneous, consisting primarily of recreationally active male individuals, and was relatively small in size. Thus, future research should examine if these similar findings can be observed within the female counterparts of similar age and activity level. Also, future research should examine if these findings remain consistent when testing sessions are performed within a longer time span and when performing other types of jump testing procedures with greater coordination demands (e.g., drop jump).

## 5 Conclusion and practical application

The results obtained during the present investigation indicate that a short-term learning effect does not impact CVJ performance within a cohort of recreationally active males across four different testing time points throughout one day. Thus, familiarization trials may not be necessary when testing the performance of movements such as CVJ, which are commonly integrated into daily physical activities. In addition, another important factor that could have contributed to the absence of the learning effect observed in the present study is the administration of adequate and standardized cues throughout the testing sessions, which allowed participants to perform CVJ movement in a more consistent manner. Overall, these findings can help strength and conditioning practitioners and sports scientists obtain a better understanding of the impact of learning effect on CVJ performance and therefore optimize the testing and training protocols for athletes and individuals engaging in similar types of physical activities.

## Data Availability

The raw data supporting the conclusions of this article will be made available by the authors, without undue reservation.
